# Validity and reliability of a questionnaire that aims to investigate consumption and problematic eating behaviours towards refined sugar

**DOI:** 10.1017/jns.2025.10051

**Published:** 2025-11-18

**Authors:** Mairi H. Gardner, John M. Grigor, Anne L. Savage, Karen L. Barton

**Affiliations:** Department of Built Environment and Life Sciences, Abertay University, Dundee, SC, UK

**Keywords:** Eating behaviours, Obesity, Refined sugar addiction, Ultra-processed food addiction, UPF, ultra-processed foods, DSM-V, Diagnostic and Statistical Manual of Mental Health Disorders, fifth edition, YFAS, Yale Food Addiction Scale, YFAS 2.0, Yale Food Addiction Scale 2.0, mYFAS, modified Yale Food Addiction Scale 2.0, HPA axis, Hypothalamic-Pituitary-Adrenal axis, EFA, exploratory factor analysis, RSCQ, refined sugar consumption questionnaire, BED, binge eating disorder, FPQ, Food preference questionnaire, BN, bulimia nervosa

## Abstract

This study aimed to develop and validate a questionnaire that investigates sugar-related eating behaviour, excessive consumption, and addictive-like eating. This questionnaire was validated using a rigorous process assessing content validity, face validity, reliability testing, feasibility testing, and construct validity. Spearman’s correlation coefficients and Cronbach’s alpha were used to assess reliability. Feasibility testing was used to further validate and confirm the scoring/categorisation of ‘low’, ‘medium’, and ‘high’ scorers for use in future research. Exploratory factor analysis and reliability analysis were used to determine underlying latent factors and assess construct validity. Content validity was assessed by health professionals (*n* = 16), face validity was assessed by the lay public who had no expertise in nutrition or addiction (*n* = 20). Reliability (*n* = 54), repeat reliability (*n* = 50), and feasibility (*n* = 113) testing were assessed with a sample from the lay public. Spearman’s correlation coefficients were in the range of 0.58–0.91 and were statistically significant (*P* < 0.001), indicating good temporal stability within the questionnaire. Cronbach’s alpha coefficients were in the range of 0.62–0.93, indicating good internal consistency. Feasibility testing confirmed the use of calculating an ‘average total score’ from the data set and splitting the data set into tertiles: low, medium, and high scorers. Exploratory factor analysis confirmed three latent factors: F1: Compulsive Eating; F2: Comfort Eating; and F3: Withdrawal. Results suggest the questionnaire is highly reliable and was successfully validated. This questionnaire can be used in research to investigate problematic and addictive-like eating behaviour and its effects on ill health.

## Introduction

Overweight and obesity have become a global epidemic, with 59% of adults considered overweight or obese.^(1)^ The rise in obesity prevalence and refined sugar consumption is intrinsically linked, with a sharp and linear rise in both sugar consumption and obesity prevalence.^(2)^ The theory of a possible ‘addiction’ to refined sugars or foods is a complex and contested idea.^(3)^ The Diagnostic and Statistical Manual of Mental Disorders (DSM-V) is a widely accepted model of ‘addiction’ or ‘substance-use disorder’. To meet the DSM-V substance-use disorder criteria, an individual must meet two or more criteria related to impaired control, social impairment, risky use, and pharmacological criteria.^(4)^ The DSM-V specifically lists nine types of substance addictions, and currently, sugar (and/or food) is not included in this list.^(4)^ Utilising the DSM-V criteria to understand addictive-like behaviours to sugars/foods is an important starting point.

‘Food Addiction’ was first hypothesised by Theron Randolph in 1956^(5)^. Since then, a large body of research has aimed to underpin the mechanisms behind a possible addictive-like consumption towards food and its highly palatable components: fat and sugar.^(5)^ Neurobiological studies conducted on animals investigate addictive-like behaviour towards sugar and observe changes in reward pathway functioning following overconsumption.^(6–9)^ Research conducted on humans also suggests an addictive-like consumption of sugar; however, the available human research is not as convincing as the animal data^(10–13)^. A central point for uncertainty and criticism of the construct of ‘food addiction’ is that food cannot be addictive as it is of core importance to survival. Therefore, a more well-informed and specific definition has emerged: ‘ultra-processed food addiction’.^(14)^ Ultra-processed foods (UPF) are ‘industrial formulations made entirely or mostly from substances extracted from foods, derived from food constituents, or synthesised in laboratories from food substrates or other organic sources’, and can be categorised by the NOVA Food Classification System.^(15)^ The NOVA system classifies foods into four groups: (1) unprocessed or minimally processed foods; (2) oils, fats, salts, and sugars; (3) processed foods; and (4) UPF.^(15)^ Examples of foods referred to as ‘ultra-processed’ include chocolate, biscuits, ice cream, energy drinks, and margarine and are often foods rich in refined sugars. There are many possible theories within the ‘food addiction’ field, yet the most likely is that refined sugars are a main driving ingredient of addictive-like consumption to UPFs, and that it is possible that the other components in UPF, i.e. fat, salt, and additives, are amplifying sugars’ palatability^(15,16)^. However, to identify the components of UPFs causing this addictive-like consumption, we must distinguish the most likely ‘culprits’ to reduce confounding variables within the research, hence the focus on refined sugars within this questionnaire. Therefore, understanding the biological and psychological responses to foods rich in refined sugars is critical to underpinning the possible addictive components of food.

As refined sugars and/or UPF are not defined as addictive, with further evidence needed to reach consensus, a harm potential model may be suggested to identify the risks associated with refined sugar consumption and subsequently how this compares to drugs of abuse. A model of ‘addiction’ or ‘substance abuse’ can be classified based on improper and disordered misuse. For example, abuse of UPF/refined sugars is commonly seen in those with a ‘reward deficient state’, those feeling difficult and complex emotions, and those who have learned problematic coping mechanisms, emotional control, and regulation through food misuse, i.e. comfort eating.^(17,18)^ Due to the highly rewarding nature of refined sugars and UPF, these foods are easily misused to appease hedonic and emotional hunger. Thus, conditioning neural reward pathways to associate palatable foods with emotional control and regulation.^(17–19)^ Research suggests that the reward pathways within the brain are activated following UPF consumption^(20)^ together with the concurrent dampening of the hypothalamic–pituitary–adrenal axis (HPA axis) that temporarily reduces the stress response.^(18–23)^ Emotional and reward-focused eating behaviours are often modelled since birth from role models and parental figures that lead to a complex and hard-to-break cycle of emotional eating.^(24)^ Evidence suggests that those who comfort eat only find solace in foods high in fat, sugar, salt, and other additives designed to be rewarding and palatable.^(20)^ Hence, suggesting an important link between unhealthy emotional regulation, comfort eating, and foods that are highly processed and rich in refined sugars.

Nutt *et al*. present a harm potential scale as a more ‘evidence-based’ method to classify addictive drugs currently classified under the Misuse of Drugs Act 1971, i.e. class A, B, C, non-classified drugs (‘A’ being the most severe).^(25,26)^ Nutt *et al*. suggest that three factors determine harm: physical (damage to organs/systems), dependence (pleasurable effects, tolerance, withdrawal), and social (cost and impact to society). Similar to tobacco and alcohol, physical harm occurs from the chronic overconsumption of sugars and UPF through an increased risk of obesity, premature morbidity, and mortality.^(25,27,28)^ A recent study by Zhao *et al*., with a total of 108,714 participants, found that higher UPF consumption was related to an increase in all-cause mortality risk.^(27)^ Dependence can also be seen in the overconsumption of UPF/refined sugars through pleasurable effects,^(29)^ repeated use to curb cravings,^(17)^ activation of the reward pathways,^(20)^ and use to appease emotional and hedonic hunger.^(18,19)^ Social implications of chronic overconsumption of UPF/refined sugars are an important factor when considering harm, and Nutt *et al*. suggest that the cost of drug or substance misuse has a considerable impact on harm. Tobacco use causes 40% of all hospital illnesses and 60% of all drug-related fatalities.^(25)^ Similarly to Tobacco, a recent report published by the UK Parliament in 2024 found that UPF consumption alone contributed to 11% of cardiovascular deaths,^(30,31)^ 9% of type 2 diabetes cases, and 12% of combined mental health disorder outcomes in the UK.^(31)^ Reducing high intakes of UPFs are estimated to reduce cardiovascular disease costs by £4 billion, type 2 diabetes costs by £3 billion,^(32)^ and the combined cost of common mental health disorders by £15 billion annually in the UK.^(33)^ Overall, a reduction of UPF consumption would reduce costs in these three disease groups by £22 billion per year in the UK, suggesting a substantial societal impact from its use and availability. UPF and foods rich in refined sugars have a high risk of potential harm, through physical, dependence, and social harm.

Currently, tools exist that assess ‘food addiction’ but do not investigate sugar or the components of food as ‘addictive’ or ‘moreish’. The Yale Food Addiction Scale (YFAS) was created in 2009 to identify ‘food addiction’ within populations and has been used globally as a tool to identify those ‘addicted’ to food.^(34)^ It was modified in 2016 to YFAS version 2.0 with thirty-five items and further shortened to mYFAS 2.0 with thirteen items.^(35,36)^ The questionnaire consists of a self-reporting tool that examines eating behaviours over ‘the past 12 months’.^(34)^ The YFAS has a good clinical validity,^(37)^ internal consistency, and convergent, discriminant, and incremental validity.^(34,38)^ However, the YFAS has limitations such as its inability to differentiate ‘food addiction’ from specific eating disorders such as binge eating disorder (BED).^(34)^ Therefore, the Refined Sugar Consumption Questionnaire (RSCQ) aims to further develop the YFAS to solely investigate refined sugars and their impact on addictive-like eating behaviour. This development is imperative to reduce the cofounding variables that we cannot see when researching ‘food’, i.e. sugar, fat, salt, additives, and their combined effect. Further structural differences can be seen from YFAS to RSCQ as the questionnaire we propose has less questions, with more response options, a lesser focus on food as a whole and binge eating disorder symptoms, with significantly different overall scoring, i.e. scoring is based on cutoff values of a total average score, not how many symptoms are met (YFAS).

The questionnaire described is an adaptation of the frequently used YFAS that focuses on refined sugar behaviours alone. It is hoped that it will provide novel and important insights into food/sugar addiction, obesity, eating disorders, and disease prevalence. The questionnaire has undergone a rigorous validation process encompassing content validity, face validity, reliability testing, feasibility testing, and construct validity. This method of validation has been used in many other questionnaire validation processes.^(34,39)^ Therefore, following validation, this questionnaire aims to provide a tool that can be used to assess sugar consumption and problematic eating behaviours towards sugar, which can be used for much-needed research in this area.

## Materials and methods

This study was conducted according to the guidelines laid down in the Declaration of Helsinki, and all procedures involving human subjects were approved by the Abertay University Ethics Committee (**EMS8811**). Written informed consent was obtained from all participants. Questionnaires on eating and food behaviour, along with those focusing on other addictive substances such as tobacco, alcohol, and recreational drugs, were reviewed for understanding and relevance, such as the ‘Food preference questionnaire for adolescents and adults’^(40)^ and the Yale Food Addiction Scale.^(41)^ Questions were curated using the DSM-V criteria for substance abuse, i.e. social impairment, pharmacological symptoms, risky use, and impaired control. A workable draft was developed and reviewed by the authors before entering the first stage of validity. The questionnaire was created using Qualtrics^XM^ online survey software (Qualtrics, Provo, UT; Copyright © 2024 Qualtrics). Participants for each validity phase were recruited randomly from a wide variety of sources, namely, through Abertay University, social media posts, posters, and word of mouth. Care was taken to ensure that the participants in each respective phase were diverse in age, ethnicity, sex, and occupation to ensure these findings are generalisable to the wider population. More specific information on participant types for each phase can be found in the results section.

### Content validity

The first phase of validity investigated the relevance and accuracy of the content of the questionnaire. This was carried out by professionals in the fields of nutrition, psychology, addiction, sociology, and food chemistry. The draft questionnaire was distributed by email to professionals. They were asked to provide feedback and scores on the relevance and importance of each question in the questionnaire. The email included a letter to the professionals and a separate document with a table that included each question and a scoring system. The scoring asked the professionals to rate each question out of 10 based on importance, appropriateness, phrasing, and overall opinion. They were also asked to provide any additional comments that they thought might be useful.

Fifteen professionals (five nutritionists, six psychologists, two addiction specialists, one sociologist, and one food chemist) provided feedback on the questionnaire, which was collated, and the questionnaire was modified accordingly.

### Face validity

Assessment of face validity of the amended questionnaire was conducted with lay individuals to assess ease of understanding and completion. Twenty individuals were asked to complete the questionnaire following written informed consent and were then interviewed regarding the completion process. Care was taken to include a range of participants with variation in age and gender. The number of interviewees (*n* = 20) was selected based on previous work indicating that at least fifteen people were sufficient to ensure a wide range of responses^(34,39)^. Those with physical and/or mental health conditions were not excluded to ensure a wide range of opinions and experiences and to increase the validity of this phase. Individuals were asked to complete the questionnaire and then answer five follow-up questions: (1) Were there any questions you did not understand? (2) Were there any words or phrases you did not understand? (3) Do you think there were any questions that were too similar/asking the same thing? (4) Did you find the response options appropriate — i.e. did you feel you could answer correctly for you? (5) Do you think the questionnaire could be improved in any way (especially in relation to ease of completion and understanding)? The time taken to complete the questionnaire was noted, their feedback was collated, and the questionnaire was modified accordingly.

### Reliability testing

Assessment of repeat reliability of the amended questionnaire following the previous two phases was undertaken with a group of adults (*n* = 54).^(34,39)^ Repeat administration of the questionnaire was carried out 1 week after the initial completion (Time 1 and Time 2). Recruitment of participants was carried out through posters, email dissemination, and social media posts throughout Abertay University. At Time 1, participants received an information sheet and were asked to complete a consent form. They were given a personalised code and asked to complete the questionnaire on two occasions, 1 week apart. A range of students, professionals, and the public participated at this stage. Spearman’s correlation coefficient analysis was undertaken for repeat reliability testing to analyse how answers to the same questions differed 1 week apart (reliability). Previous work indicated that each question must be significant (*p* < 0.05) and have a correlation coefficient of >0.5 to be considered reliable.^(39,42)^ Cronbach’s alpha coefficients were computed to test the internal consistency of similar grouped questions on Time 1 data. Cronbach’s alpha values of >0.70 are considered satisfactory for inclusion.^(43)^ There were some minor changes needed to some of the questions, which were identified after the reliability testing phase such as changes to scoring and wording of certain questions due to inconsistencies and conciseness. Therefore, a further ten people (lay public) were recruited to assess face validity on these questions alone. Minor changes were made following feedback.

### Feasibility testing

Following the second face validity phase, a further ninety participants were sought to complete the questionnaire to assess its usability with a larger sample size. G Power was used to calculate an adequate sample size with an effect size of 0.459. This effect size was estimated based on a univariate ANOVA calculation to estimate effect size from responses to the final question (‘I think I am addicted to sugar’), thus suggesting a sample size of 90. Participants were recruited through the university, social media, and posters, and a final sample of 113 participants was collected. Participants were ranked from lowest to highest based on their average total score and split into tertiles to form low, medium, and high scorers. The minimum score possible is 0, and the maximum score possible is 116. Other researchers have used this sample-specific method when using the ‘Energy-Adjusted Dietary Inflammatory Index’ to understand inflammation, stress, and anxiety in university students.^(44)^ The sample is divided into three tertiles based on the distribution of scores and impacts investigated within these three tertiles.^(44)^ Furthermore, White *et al*. used the ‘Perceived Stress Scale’ to investigate stress and pain in older adults and used a sample-specific approach to split the data into three tertiles based on their own data set.^(45)^ A Monte Carlo simulation analysis was also carried out in R Studio to assess the usability of these cutoff values in a simulated sample size of 10,000.^(46)^

## Results

### Content validity

Fifteen professionals (five nutritionists, six psychologists, two addiction specialists, one sociologist, and one food chemist) responded and provided feedback regarding a request for comments. Amendments to the initial questionnaire draft were considered based on the feedback provided by professionals. Many of the amendments were made due to concerns about interpretation. This resulted in the simplification and/or separation of certain questions, especially questions that may be asking about two related concepts. There were also slight amendments to the wording or phrasing of questions. Questions were also amended so that important symptoms of addiction could be considered, such as ‘consumption to exhaustion’ and ‘drug seeking behaviours’. Questions relating to these concepts were added, such as eating sugary foods and/or drinks ‘until they are all gone’ and ‘buying sugary foods or drinks as soon as they run out’. These specific amendments were suggested by addiction specialists. Two questions were added regarding ‘social impairment’ (a DSM-V criterion) as professionals felt that this was missing, as well as two questions regarding opinions on sugar consumption and ‘addiction’, i.e. ‘I think I am addicted to sugar’ and ‘My sugar consumption is higher than most people I know’. Three questions were removed as feedback suggested they were too similar/not required.

To improve the flow and structure, the questionnaire was reordered. Demographic and consumption questions were added towards the end of the questionnaire, whilst questions relating to the DSM-V criteria (cravings, withdrawal) were moved to the beginning.

### Face validity

Twenty adults (eleven females, nine males), with an age range of 20–66 years, completed the questionnaire and were subsequently interviewed. The typical completion time was 4–14 min (mean 8 min ± S.D. 3.31). Minor adjustments were made to the questionnaire to improve clarity of responses and interpretation of questions. Two questions were added to determine if the participant had any mental health conditions (i.e. generalised anxiety disorder, eating disorders) or if they took any medications that may influence eating behaviour. Minor structural changes such as adding headings to sections were also made. A question was added at the end of the questionnaire asking the participant if they wanted to provide any further information on their sugar consumption (via a textbox) for further elaboration. Following feedback from several participants, the ‘neither agree nor disagree’ option was considered in detail^(47,48)^. Many participants suggested that they used this response when they ‘didn’t know’ or were ‘unsure’. Following consideration, the ‘neither agree nor disagree’ option was retained, due to the importance of a ‘neutral’ point in Likert scales and questionnaire creation.^(47,48)^ However, clarification was added at the beginning of the questionnaire on when participants should use ‘neither agree nor disagree’ as a response, and an ‘unsure/I don’t know’ option was added to each question to ensure participants could answer appropriately.

### Reliability testing

Participants were asked to complete the questionnaire on two separate occasions, 1 week apart (Time 1 was the first completion, and Time 2 was the second completion). Fifty-four individuals completed the questionnaire at Time 1. Of those, sixteen respondents were male, thirty-seven were female, and one person identified as non-binary. Ages ranged from 18 to 66 years (mean 39.0 years ± S.D. 15.2). Fifty individuals completed the questionnaire at Time 2 (four individuals surpassed the week time limit and were therefore excluded from correlation analysis). The fifty respondents comprised fourteen males, thirty-five females, and one non-binary person with an age range of 18–66 years (mean 39.36 years ± S.D. 15.2). All questions were fully completed. Respondents were predominantly Caucasian, with three participants of African American, Thai, and Filipino ethnicities. The sample was evenly distributed across the Scottish Index of Multiple Deprivation deciles.^(49)^

Cronbach’s alpha coefficients were computed on Time 1 data due to the higher number of respondents and less risk of external contamination than with Time 2 data. The Cronbach’s alpha of each section is presented in Table 1. Repeat-reliability testing was carried out on the data for the fifty respondents who completed the questionnaire at Time 1 and Time 2. Spearman correlation coefficients were in the range of 0.58–0.91 and were statistically significant (*P* < 0.001), indicating good temporal stability (Table 2).

**Table 1.** Cronbach’s alpha to assess internal consistency (*n* = 54)

Questions	Cronbach’s alpha
Q1. I have cravings for sugary foods and/or drinks. Q12. If I run out of sugary foods and/or drinks, I will go out and buy more as soon as possible. Q13. When I eat fewer sugary foods and/or drinks, I have stronger urges to consume them. Q30. I think I am addicted to sugar.	0.728
Q5. When there are sugary foods and/or drinks available to me, I eat them straight away or constantly think about eating them. Q6. I find it hard to stop eating sugary foods and/or drinks once I start. Q7. I find it impossible to stop eating sugary foods and/or drinks and will not stop until they are all gone. Q8. I binge eat sugary foods and/or drinks and eat/drink a lot in one sitting. Q9. I eat/drink sugary foods and/or drinks to the point where I feel physically ill.	0.875
Q15. I want to eat sugary foods and/or drinks when I feel negative emotions such as anxiety, sadness, or depression. Q16. I consume sugary foods and/or drinks to ease feelings of anxiety or stress. Q17. I consume sugary foods and/or drinks to ease feelings of depression.	0.893
Q18. I have emotional withdrawal symptoms such as anxiety or agitation when I cut down or stop eating sugary foods and/or drinks. Q19. I have physical withdrawal symptoms such as a headache, tiredness, or physical pain when I cut down or stop eating sugary foods and/or drinks.	0.831
Q15. I want to eat sugary foods and/or drinks when I feel negative emotions such as anxiety, sadness, or depression. Q16. I consume sugary foods and/or drinks to ease feelings of anxiety or stress. Q17. I consume sugary foods and/or drinks to ease feelings of depression. Q18. I have emotional withdrawal symptoms such as anxiety or agitation when I cut down or stop eating sugary foods and/or drinks. Q19. I have physical withdrawal symptoms such as a headache, tiredness, or physical pain when I cut down or stop eating sugary foods and/or drinks. Q20. My sugar consumption causes me to feel guilty, and yet I can’t cut down on sugary foods and/or drinks. Q21. My sugar consumption causes me to feel self-loathing, and yet I can’t cut down on sugary foods and/or drinks. Q22. My sugar consumption causes me to feel depressed, and yet I can’t cut down on sugary foods and/or drinks. Q23. My sugar consumption causes me to feel anxiety, and yet I can’t cut down on sugary foods and/or drinks.	0.918
Q19. My sugar consumption causes me to feel guilty, and yet I can’t cut down on sugary foods and/or drinks. Q20. My sugar consumption causes me to feel self-loathing, and yet I can’t cut down on sugary foods and/or drinks. Q21. My sugar consumption causes me to feel depressed, and yet I can’t cut down on sugary foods and/or drinks. Q22. My sugar consumption causes me to feel anxiety, and yet I can’t cut down on sugary foods and/or drinks.	0.931
Q28. When in social occasions, do you persuade others to get a dessert/sweet treat even when they don’t want one, so you can have one? Q29. When eating out, and you want dessert, do you get frustrated at those with you if they don’t want a dessert?	0.621

**Table 2.** Spearman’s correlation coefficient for repeat reliability test/retest (*n* = 50)

Question number	Question label	Test-retest reliability*
Q1	I have cravings for sugary foods and/or drinks.	0.81
Q2	I want to cut down on sugary foods and/or drinks.	0.91
Q3	I struggle to cut down on sugary foods and/or drinks.	0.69
Q4	Eating too many sugary foods and/or drinks is something I worry about.	0.61
Q5	When there are sugary foods and/or drinks available to me, I eat them straight away or constantly think about eating them.	0.70
Q6	I find it hard to stop eating sugary foods and/or drinks once I start.	0.80
Q7	I find it impossible to stop eating sugary foods and/or drinks and will not stop until they are all gone.	0.81
Q8	I binge eat sugary foods and/or drinks and eat/drink a lot in one sitting.	0.70
Q9	I eat/drink sugary foods and/or drinks to the point where I feel physically ill.	0.76
Q10	Please complete the sentence below with the most appropriate answer. When sugary foods and/or drinks are offered to me, I can resist them…	0.91
Q11	Please complete the sentence below with the most appropriate answer. When there are sugary foods and/or drinks on display, I can resist buying them…	0.58
Q12	If I run out of sugary foods and/or drinks, I will go out and buy more as soon as possible.	0.61
Q13	When I eat fewer sugary foods and/or drinks, I have stronger urges to consume them.	0.73
Q14 *	I comfort eat sugary foods and/or drinks.	
Q15	I want to eat sugary foods and/or drinks when I feel negative emotions such as anxiety, sadness, or depression.	0.76
Q16	I consume sugary foods and/or drinks to ease feelings of anxiety or stress.	0.73
Q17	I consume sugary foods and/or drinks to ease feelings of depression.	0.64
Q18	I have emotional withdrawal symptoms such as anxiety or agitation when I cut down or stop eating sugary foods and/or drinks.	0.77
Q19	I have physical withdrawal symptoms such as a headache, tiredness, or physical pain when I cut down or stop eating sugary foods and/or drinks.	0.59
Q20	My sugar consumption causes me to feel guilty, and yet I can’t cut down on sugary foods and/or drinks.	0.66
Q21	My sugar consumption causes me to feel self-loathing, and yet I can’t cut down on sugary foods and/or drinks.	0.86
Q22	My sugar consumption causes me to feel depressed, and yet I can’t cut down on sugary foods and/or drinks.	0.84
Q23	My sugar consumption causes me to feel anxiety, and yet I can’t cut down on sugary foods and/or drinks.	0.72
Q24	I eat biscuits, cakes, chocolate, sweets, desserts, and other sugary foods.	0.82
Q25	I drink beverages with added sugar, i.e. full sugar Coke (not ‘diet’ alternatives with added sweetener).	0.77
Q26	Do you feel stressed regularly (any change or event that causes continuous ’physical, emotional, or psychological strain’, i.e. work-related stress)?	0.87
Q27	I believe my sugar consumption is higher than most other people I know.	0.76
Q28	When in social occasions, do you persuade others to get a dessert/sweet treat even when they don’t want one, so you can have one?	0.77
Q29	When eating out, and you want dessert, do you get frustrated at those with you if they don’t want a dessert?	0.68
**	Choose the answer that applies most from the list below (sweet or savoury tooth question).	0.87
Q30	I think I am addicted to sugar.	0.80

* Question added after analysis. ** Question removed after analysis.

Minor changes were made to the questionnaire before the final phase of validity, i.e. the phrasing of certain questions and minor changes to the Likert scale on two questions. The draft was amended before the next phase.

### Feasibility testing

The scale on two questions (Q1 and Q2) was changed from ‘Yes/No’ to ‘Strongly Agree to Strongly Disagree’ as this was deemed more appropriate for the scoring and matched the other Likert scales. Another two questions were added, i.e. ‘I comfort eat sugary foods and/or drinks’ and ‘Do you have any mental (i.e. anxiety, depression) or physical (i.e. asthma, diabetes) health conditions? Please specify which condition(s)’. Following these changes, 113 participants were recruited to complete the feasibility testing phase. Of those 113 participants, 76 were female, 34 were male, 2 were general neutral, and 1 identified as a third gender. Ages ranged from 18 to 69 years (mean 37.8 years ± S.D. 14.7). See Table 3 for more information. The sample was predominantly White (*n* = 99) with three Black African, two Middle Eastern, four Indian, four other, and one who preferred not to say. The main aim of this phase was to confirm the scoring, validity, and usability of the questionnaire within a powered sample size. Section 3.5 discusses the scoring in more detail.

**Table 3.** Descriptive statistics on total scores/average scores *n* = 113

	Total score	Average score
**Mean**	46.3	1.6
**Median**	46	1.6
**Mode**	21	0.7
**Range**	110.5	3.8
**Standard deviation**	20.3	0.7

### Scoring

Scoring of the questionnaire was important to consider for the usefulness of the questionnaire in future research. Many possible scoring avenues were considered following the development and validation of the questionnaire. It was decided to establish an average total score. Each question was scored from 0–4. Question 26 ‘Do you feel stressed regularly (any change or event that causes continuous ‘physical, emotional or psychological strain’, i.e. work-related stress)?’ received no score as stress levels do not directly correlate with sugar consumption and/or eating behaviours and therefore may skew the total score. Please see Table 4 for specific scoring instructions. Total scores were calculated for each participant, and an average score of the questionnaire responses was calculated to account for those who answered some questions with neutral response options, i.e. ‘Unsure/I don’t know’ and/or ‘Prefer not to say’ as these responses received no score. This may have skewed the data in participants who answered questions with these responses and may not be representative of their true score. Therefore, an average of all question scores was calculated to account for this issue (sum of all scores ÷ divided by the number of scored questions = total average score). Of the 113 participants who completed the questionnaire, the participant with the lowest total score was 1.5, and the highest was 112. The tertile boundaries for this data set (*n* = 113) based on the participants’ total **average** score were first (low score) 0.05–1.26, second (medium score) 1.27–1.93, and third (high score) 1.94–3.86. The average score cutoffs are then used to group participants as ‘low’, ‘medium’, or ‘high’ scorers. The ‘Unsure/I don’t know’ or ‘Prefer not to say’ responses receive no score and, when averaged, do not influence the final total score. The average score is calculated for all participants, regardless of response options. It is important to consider the effect of these responses on data loss and the impact on statistical analysis. Out of all the responses for each participant and question (*n* = 3277), only fifty-nine responses were ‘Unsure/I don’t know’ (*n* = 58) or ‘Prefer not to say’ (*n* = 1), which suggests, within this sample, only 1.8% of the responses were non-scored options. Therefore, the data loss in this sample is not statistically meaningful.

**Table 4.** Question scoring

Question number	Question label	Scoring
Q1	I have cravings for sugary foods and/or drinks.	0–4
Q2	I want to cut down on sugary foods and/or drinks.	0–4
Q3	I struggle to cut down on sugary foods and/or drinks.	0–4
Q4	Eating too many sugary foods and/or drinks is something I worry about.	0–4
Q5	When there are sugary foods and/or drinks available to me, I eat them straight away or constantly think about eating them.	0–4
Q6	I find it hard to stop eating sugary foods and/or drinks once I start.	0–4
Q7	I find it impossible to stop eating sugary foods and/or drinks and will not stop until they are all gone.	0–4
Q8	I binge eat sugary foods and/or drinks and eat/drink a lot in one sitting.	0–4
Q9	I eat/drink sugary foods and/or drinks to the point where I feel physically ill.	0–4
Q10	Please complete the sentence below with the most appropriate answer. When sugary foods and/or drinks are offered to me, I can resist them…	4–0
Q11	Please complete the sentence below with the most appropriate answer. When there are sugary foods and/or drinks on display, I can resist buying them…	4–0
Q12	If I run out of sugary foods and/or drinks, I will go out and buy more as soon as possible.	0-4
Q13	When I eat fewer sugary foods and/or drinks, I have stronger urges to consume them.	0–4
Q14 *	I comfort eat sugary foods and/or drinks	0–4
Q15	I want to eat sugary foods and/or drinks when I feel negative emotions such as anxiety, sadness, or depression.	0–4
Q16	I consume sugary foods and/or drinks to ease feelings of anxiety or stress.	0–4
Q17	I consume sugary foods and/or drinks to ease feelings of depression.	0–4
Q18	I have emotional withdrawal symptoms such as anxiety or agitation when I cut down or stop eating sugary foods and/or drinks.	0–4
Q19	I have physical withdrawal symptoms such as a headache, tiredness, or physical pain when I cut down or stop eating sugary foods and/or drinks.	0–4
Q20	My sugar consumption causes me to feel guilty, and yet I can’t cut down on sugary foods and/or drinks.	0–4
Q21	My sugar consumption causes me to feel self-loathing, and yet I can’t cut down on sugary foods and/or drinks.	0–4
Q22	My sugar consumption causes me to feel depressed, and yet I can’t cut down on sugary foods and/or drinks.	0–4
Q23	My sugar consumption causes me to feel anxiety, and yet I can’t cut down on sugary foods and/or drinks.	0–4
Q24	I eat biscuits, cakes, chocolate, sweets, desserts, and other sugary foods.	0–4 (0.5, 1, 1.5, 2, 2.5, 3, 3.5, 4)
Q25	I drink beverages with added sugar, i.e. full sugar Coke (not ‘diet’ alternatives with added sweetener).	0–4 (0.5, 1, 1.5, 2, 2.5, 3, 3.5, 4)
Q26	Do you feel stressed regularly (any change or event that causes continuous ‘physical, emotional, or psychological strain’, i.e. work-related stress)?	0 (no score)
Q27	I believe my sugar consumption is higher than most other people I know.	0–4
Q28	When in social occasions, do you persuade others to get a dessert/sweet treat even when they don’t want one, so you can have one?	0–4
Q29	When eating out, and you want dessert, do you get frustrated at those with you if they don’t want a dessert?	0–4
Q30	I think I am addicted to sugar.	0–4

* Options ‘Unsure/ I don’t know’ or ‘Prefer not to say’ receive no score (0).

**Fig. 1.**
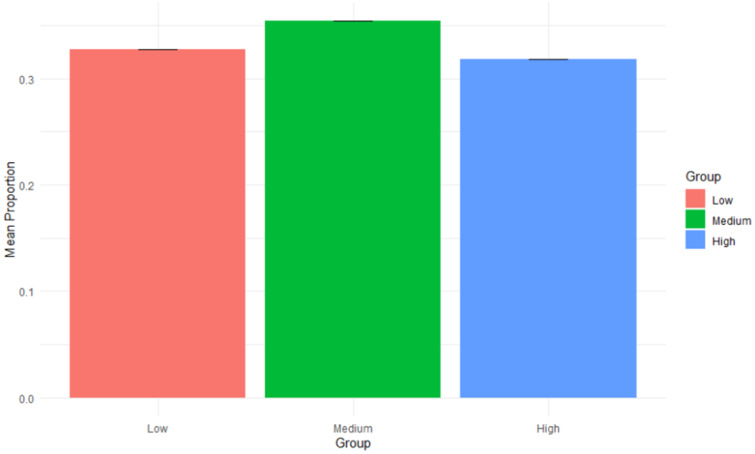
Monte Carlo Simulation – Mean Proportions by group with 95% confidence intervals.

Furthermore, to evaluate the use of these cutoff values and a sample-specific approach, a Monte Carlo simulation analysis was performed. This analysis relies upon repeated random sampling to predict potential outcomes.^(50,51)^ In this case, R Studio was used to simulate the data from 113 participants at a population size of 10,000.^(46)^ Mean proportions and 95% CIs can be seen below in Figure 1. In this simulation, the low group had a mean proportion of 32.7%, the medium group had a mean proportion of 35.4%, and the high group had a mean proportion of 31.9%, suggesting that most participants fall into the middle category yet are well distributed between all three groups. This suggests that these groups are consistent and reliable when scaled to a larger sample size and that the current data set (n = 113) is representative of the population.

### Exploratory factor analysis

**Fig. 2.**
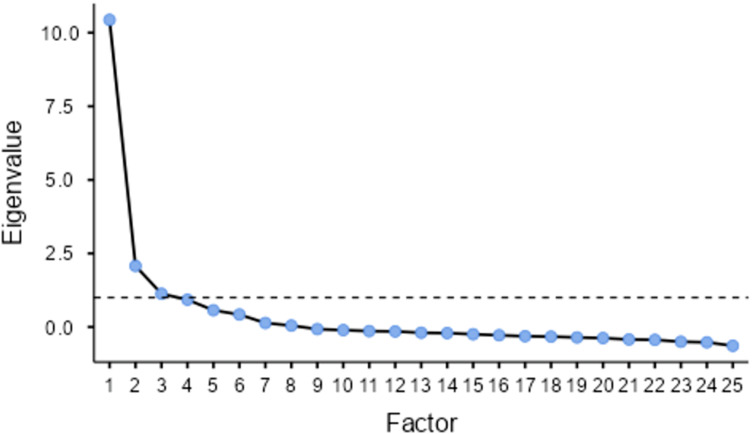
Scree plot.

Exploratory factor analysis (EFA) was carried out to investigate the underlying latent factors within the questionnaire items, which allows us to identify unknown factors and constructs that ensure the intended variables are being measured.^(52)^ The EFA also allows us to assess internal reliability and dimensionality and is commonly used when validating measurement instruments such as questionnaires.^(52)^ The EFA used the minimum residuals extraction method with oblimin rotation as the items within the questionnaire are correlated and may overlap. The analysis suggests that there were three latent factors within the questionnaire data. The factor loadings were set to >0.4, and eigenvalues above 1 were considered. As seen in Table 5 and Figure 2, Factor 1 consisted of questions 1–8, 10–12, 20–24, and Q30, with the lowest values of uniqueness (suggesting the factor is well explained by these items) on Q8, and Q5, 20, 21, and 23. These questions (among the rest in this factor) are related to compulsive eating, with a specific theme of inability to stop eating/drinking sugary foods or drinks. Factor 2 consisted of questions 7–8 and 14–17, with the lowest value of uniqueness on Q16, Q14, Q15, and Q17. These questions relate to comfort eating, and particularly eating sugary foods and/or drinks to ease feelings such as stress, anxiety, depression, and sadness. Finally, Factor 3 consisted of questions 13, 18, 19, and 27, with the lowest value of uniqueness on Q18 and Q19, which consisted of questions on emotional and physical withdrawal. Therefore, the three latent factors are defined as F1: Compulsive Eating, F2: Comfort Eating, and F3: Withdrawal. Overall, the factors represented 57.5% of the total variance. A further reliability analysis was run in Jamovi to assess the internal consistency of each factor. F1 had a Cronbach’s alpha of 0.929, F2 with 0.924, and F3 with 0.851, thus suggesting that the items within each factor were highly correlated and measured the same underlying constructs.

**Table 5.** Exploratory factor analysis

Item	Factor 1	Factor 2	Factor 3	Uniqueness
Q1 (I have cravings for sugary foods and/or drinks.)	0.613			0.474
Q2 (I want to cut down on sugary foods and/or drinks.)	0.607			0.694
Q3 (I struggle to cut down on sugary foods and/or drinks.)	0.713			0.410
Q4 (Eating too many sugary foods and/or drinks is something I worry about.)	0.626			0.660
Q5 (When there are sugary foods and/or drinks available to me, I eat them straight away or constantly think about eating them.)	0.801			0.352
Q6 (I find it hard to stop eating sugary foods and/or drinks once I start.)	0.508			0.508
Q7 (I find it impossible to stop eating sugary foods and/or drinks and will not stop until they are all gone.)	0.471	0.404		0.446
Q8 (I binge eat sugary foods and/or drinks and eat/drink a lot in one sitting.)	0.436	0.428		0.317
Q10 (Please complete the sentence below with the most appropriate answer. When sugary foods and/or drinks are offered to me, I can resist them.)	0.680			0.624
Q11 (Please complete the sentence below with the most appropriate answer. When there are sugary foods and/or drinks on display, I can resist buying them.)	0.636			0.659
Q12 (If I run out of sugary foods and/or drinks, I will go out and buy more as soon as possible.)	0.504			0.668
Q13 (When I eat fewer sugary foods and/or drinks, I have stronger urges to consume them.)			0.602	0.553
Q14 (I comfort eat sugary foods and/or drinks.)		0.814		0.270
Q15 (I want to eat sugary foods and/or drinks when I feel negative emotions such as anxiety, sadness, or depression.)		0.871		0.180
Q16 (I consume sugary foods and/or drinks to ease feelings of anxiety or stress.)		0.882		0.137
Q17 (I consume sugary foods and/or drinks to ease feelings of depression.)		0.748		0.223
Q18 (I have emotional withdrawal symptoms such as anxiety or agitation when I cut down or stop eating sugary foods and/or drinks.)			0.953	0.118
Q19 (I have physical withdrawal symptoms such as a headache, tiredness, or physical pain when I cut down or stop eating sugary foods and/or drinks.)			0.863	0.245
Q20 (My sugar consumption causes me to feel guilty, and yet I can’t cut down on sugary foods and/or drinks.)	0.612			0.320
Q21 (My sugar consumption causes me to feel self-loathing, and yet I can’t cut down on sugary foods and/or drinks.)	0.580			0.336
Q22 (My sugar consumption causes me to feel depressed, and yet I can’t cut down on sugary foods and/or drinks.)	0.512			0.491
Q23 (My sugar consumption causes me to feel anxiety, and yet I can’t cut down on sugary foods and/or drinks.)	0.505			0.398
Q24 (I eat biscuits, cakes, chocolate, sweets, desserts, and other sugary foods.)	0.619			0.679
Q27 (I believe my sugar consumption is higher than most other people I know.)			0.500	0.492
Q30 (I think I am addicted to sugar.)	0.539			0.382

* Minimum residual’ extraction method was used in combination with an ‘oblimin’ rotation.

**Questions 9, 25, 28, and 29 were removed from the analysis as they appeared on no loadings.

It is important to address whether the Cronbach’s alpha analysis carried out on the reliability phase (*n* = 54) correlates with the exploratory factor analysis presented here. Please see Table 1 for the Cronbach’s alpha analysis carried out in the reliability phase. In this analysis, seven groupings of similar questions were identified, and all groups had good internal consistency and a Cronbach’s alpha range of 0.62–0.93. The EFA grouped many of the questions on the same factors; however, there was a slight overlap between factors when comparing these two analyses. The EFA more precisely allocates the questions to the three underlying factors. This is to be expected due to the complex and overlapping pathology of eating behaviours, particularly of emotional and addictive-like eating.

## Discussion

This questionnaire was developed and validated based on DSM-V and YFAS that score individuals specifically on their sugar consumption and sugar-related eating behaviours. This questionnaire is an important tool and future step to categorising and investigating complex individual relationships towards sugary foods and its impact on health and obesity. Furthermore, future research is needed to understand the addictive-like consumption of UPFs and the hypothesis that refined sugar is a key driver behind UPF addiction. Currently, 14% of adults^(53)^ and 15% of youths^(54)^ are classified as ‘food addicts’.^(53,54)^ The rates of substance-use disorders are notably similar to the rate of UPF ‘addiction’ with alcohol-use disorder at 14% and tobacco-use disorder at 18%.^(55,56)^ The NOVA food classification system can provide some clarity on what foods are defined as ‘ultra-processed’. However, there is some debate on its effectiveness and practicality, as there is little consensus on what foods classify as ‘ultra-processed’ and if processing always leads to poorer nutrient quality.^(57)^ Nevertheless, when foods are classified as ‘ultra-processed’, they almost always have one major component: refined sugars. Refined sugars may be a major driver behind ‘UPF addiction’ such as nicotine in tobacco or ethanol in alcohol.^(58,59)^ Regular consumption of UPF has been suggested to increase the risk of being overweight and obese.^(60,61)^ Therefore, there may be a complex, yet critical relationship between obesity, UPF, refined sugar intake, and addictive-like behaviours. Hall et al. found that those who consume a diet rich in UPF on average consume an extra 500 calories per day, rich in fat and sugars (but not protein), than those eating an unprocessed diet.^(62)^ Current interventions to assess or investigate obesity within populations are unsuccessful and/or low in impact, with the concept of ‘refined sugar addiction’ and/or ‘UPF addiction’ less explored, despite the body of research that suggests its plausibility. This factor is critical to investigate when assessing the obesity epidemic and a tool to understand unhealthy food behaviours.

Currently, the YFAS is the main tool to assess ‘food addiction’; this tool is critical but not suitable to assess the construct of ‘sugar addiction’ or assess refined sugar consumption. The YFAS may struggle to differentiate ‘food addiction’ from other eating disorders such as BED or bulimia nervosa (BN), and this may be considered a limitation of the scale. Gearhardt *et al.* recorded the highest prevalence of ‘food addiction’ among individuals with BED at 56.8%, with the highest mean YFAS symptoms at 4.6 out of a possible 7.^(38)^ The prevalence of YFAS diagnosis was found to be greater still in those with BN, with 83.6% meeting the YFAS for ‘food addiction’.^(63)^ BED possesses many characteristics of addictive behaviours, such as diminished control and continued use despite negative consequences.^(64)^ Despite their similarities, BED and UPF/food addiction represent comparable, yet distinctly different conditions.^(64)^ The ‘Refined Sugar Consumption Questionnaire’ described in this paper aims to identify behaviours associated with sugar consumption alone, separately from other eating behaviours. It is hypothesised that those with BED or BN will receive higher scores, yet this tool may be able to differentiate between problematic behaviours associated with sugar and eating disorders due to such a rigorous development and validation process with this limitation in mind, while removing the focus on eating behaviours and food consumption as a whole.

**Fig. 3.**
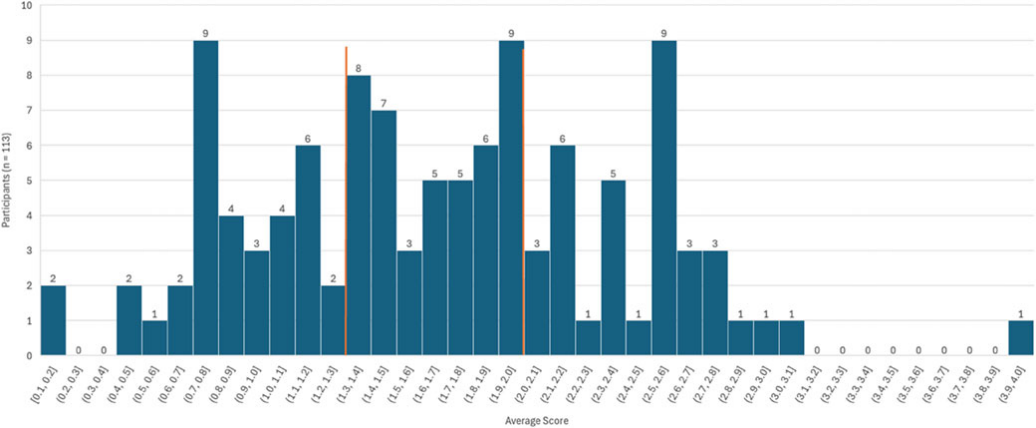
Average score distribution.

The usability of this questionnaire in future research is vital, and creating a scoring system to group participants into low, medium, and high was necessary. Figure 3 shows the distribution of the average scores in this sample. The orange lines represent the score cutoff points for low, medium, and high groups. Most of the participants were categorised in the medium group, with fewer participants in the low and high groups. Only one participant had a very low score of 1.5 (average score of 0.05), and one had a very high score of 112 (average score of 3.86), thus suggesting a normally distributed sample. Exploring the use of the questionnaire in clinical and epidemiological settings provides a valuable future direction, and further refinement to develop it for use in clinical settings is essential. The consensus on ‘refined sugar addiction’ and ‘ultra-processed food addiction’ remains controversial, and a clinical definition or consensus has not yet been reached. It is therefore difficult to suggest clinical applications for the questionnaire. However, identifying addictive-like tendencies to refined sugars is important when considering emotional eating, disordered eating, and applications for obesity management and prevention in clinical settings. Further work to develop and review this questionnaire for use in clinical settings is important, but first, we must carry out further research on definitions, plausibility, consequences, and shifts towards addictive-like eating management and prevention in clinical settings.

Furthermore, utilising the DSM-V criterion to assess refined sugar ‘addiction’ is an important starting point when considering clinical and social implications and applications of UPF/refined sugar ‘addictions’. However, further research and development of the DSM-V criteria are needed to assess a substance such as food as ‘addictive’ due to its complex nature. Currently, the DSM-V was created and exists to investigate drugs of abuse that are human-made substances and are not dependent or necessary for survival. Food is vital for survival, although there is some debate on whether we can define UPF/refined sugars as food as they are so far removed from their original food constituents and are null of nutrients.^(65)^ However, it cannot be ignored that it is nearly impossible to avoid refined sugars and UPF in the current westernised food environment. We must consider this and investigate addictive-like consumption of foods as a branch of addictive-like and compulsive behaviours that are separate from drugs of abuse as they are often illegal, non-food stuffs, and human synthesised. Further longitudinal, neurobiological, and clinical studies investigating UPF and refined sugar ‘addictions’ are necessary to support this addition and potential development to the DSM-V to include certain foods. In the case of UPFs, we cannot simply add these substances to the list of known addictions (following adequate research) due to their complexity and the integral (yet problematic) part they play in westernised society. An approach to classify certain foods as ‘addictive’ would likely take a different format than the currently known addictions. This should become clearer with further research conducted on refined sugar and UPF addiction in humans.

To validate this questionnaire, the use of an independent panel of experts to assess the content was important and a common starting point for questionnaire design.^(39,66–68)^ Therefore, the complexities of the questionnaire were assessed by each respective professional. When assessing the validity and effectiveness of a tool that will be primarily disseminated to the public, it is vital that comprehensibility and ease of completion are assessed. This questionnaire was written with the lay individual in mind and created for individuals to complete without the help or advice from a professional. Of sixty articles assessing food addiction via the YFAS, thirty of the studies used an online version of the questionnaire, without an interviewer present, and the others were carried out in clinics or laboratories with a professional.^(69)^ The described questionnaire can be completed by a lay individual in a community setting and could also be used in clinics or laboratories. This ensures the use of this tool is wide-ranging to investigate sugar consumption and health.

The reliability and validity testing undertaken here does not guarantee that the tool is suitable in all populations, i.e. all regions of the UK, ethnic minorities, all age groups, or those who are illiterate. However, the questionnaire validation process included a wide range of participants and was created with these factors in mind. In the reliability testing phase, it was assumed that participants were not influenced by the first exposure to the questionnaire, i.e. prompted to change their behaviours. Participants were not subjected to any interventions between Time 1 and Time 2, and therefore, there is no reason to think that participants changed their behaviour within the week. However, it is possible that participants may have been motivated to change behaviours in relation to their sugar consumption after the first exposure to the questionnaire due to the exploratory nature of the questions. However, it is unlikely that a notable change occurred within the week and before the second exposure to the questionnaire.

It is important to note that the reliability and validity procedure undertaken here is for the complete set of items, and if any new questions are added or elements of the questionnaire are changed, the questionnaire should undergo further validation testing. The questionnaire can be used in many settings, particularly within the UK; however, it is noted that validation was not completed in other countries with different primary languages and may not be suitable for other populations/countries. A validation process should be undertaken if this questionnaire were to be disseminated in other countries/languages or utilised for specific populations. Further feasibility testing in different settings may be useful to test the usability of the questionnaire in different populations or within populations with learning disabilities and/or literacy problems.

## Conclusion

The YFAS and the DSM-V criteria were used in conjunction to create a specific tool investigating sugar consumption alone, i.e. the ‘Refined Sugar Consumption Questionnaire’. The final questionnaire comprises thirty-one questions with the aim of investigating sugar consumption within populations (see supplementary material for full questionnaire). Validation of the questionnaire was achieved using a five-step process containing all important aspects of validity. Repeat-reliability results were strongly correlated, which suggested the reliability of the tool to assess sugar consumption and behaviour. The long-term aim of this questionnaire is to serve as a tool to investigate sugar behaviour and sugar ‘addiction’ by highlighting ‘at-risk populations’ to sugar addiction, i.e. those with high scores. Further evidence on food behaviours such as addictive-like consumption is important, including a focus on how food ‘addictions’ differ from other addictive drugs. This difference should be considered as an important distinction instead of including ‘food addiction’ into the DSM-V in the same capacity as illegal class A drugs. A proposed different outlook and category for foods should be considered. This questionnaire will aid in this needed future human research on the phenomenon of ‘refined sugar addiction’, sugar consumption, and health and may narrow the widening gap of understanding between obesity, sugar consumption, and ill health, thus providing clarity on the true nature of UPFs and refined sugars and adopting new understandings and methods to categorise such substances and behaviours.

## Supporting information

Gardner et al. supplementary material Gardner et al. supplementary material

## Supplementary material

The supplementary material for this article can be found at https://doi.org/10.1017/jns.2025.10051.
