# Supplementary material for: Validity and reliability of a questionnaire that aims to investigate consumption and problematic eating behaviours towards refined sugar
Source: J Nutr Sci. 2025 Nov 18;14:e80. doi: 10.1017/jns.2025.10051 (PMC12658297; doi:10.1017/jns.2025.10051)
Supplement: Gardner et al. supplementary material [file S2048679025100517sup001.pdf]

Refined Sugar Consumption Questionnaire

When answering the questions below please refer to this definition: ‘Sugary foods and drinks' refers to foods and drinks that are high in refined sugars for example, chocolate, sweets, cakes, biscuits and sugar-containing soft drinks.

Please answer all the questions below and select the response that applies to you.

Please note that the response "Neutral / Neither agree nor disagree" refers to a 'neutral' position i.e. when you feel you do not have an opinion OR choose "Unsure/I don't know" if you are unsure or don't know.

All questions require a response. If you would prefer not to say, please state this.

The following questions refer to the past 12 months. When answering, think about your answer in relation to this time frame.

There are no right or wrong answers, please answer as honestly as possible and remember, your responses are completely anonymous.

I have cravings for sugary foods and/or drinks.

- ○ Never
- ○ Rarely
- ○ Sometimes
- ○ Often
- ○ Always
- ○ Unsure / I don't know
- ○ Prefer not to say

I want to cut down on sugary foods and/or drinks.

- ○ Never
- ○ Rarely
- ○ Sometimes
- ○ Often

- Always
- Unsure / I don't know
- Prefer not to say

I struggle to cut down on sugary foods and/or drinks.

- Strongly disagree
- Disagree
- Neutral / Neither agree nor disagree
- Agree
- Strongly agree
- Unsure / I don't know
- Prefer not to say

Eating too many sugary foods and/or drinks is something I worry about.

- Never
- Rarely
- Sometimes
- Often
- Always
- Unsure / I don't know
- Prefer not to say

When there are sugary foods and/or drinks available to me I eat them straight away or constantly think about eating them.

- Never
- Rarely
- Sometimes
- Often
- Always
- Unsure / I don't know
- Prefer not to say

I find it hard to stop eating sugary foods and/or drinks once I start.

- Never
- Rarely
- Sometimes
- Often
- Always
- Unsure / I don't know
- Prefer not to say

I find it impossible to stop eating sugary foods and/or drinks and will not stop until they are all gone.

- Never
- Rarely
- Sometimes
- Often
- Always
- Unsure / I don't know
- Prefer not to say

I binge eat sugary foods and/or drinks and eat/drink a lot in one sitting.

- Never
- Rarely
- Sometimes
- Often
- Always
- Unsure / I don't know
- Prefer not to say

I eat/drink sugary foods and/or drinks to the point where I feel physically ill.

- Never
- Rarely
- Sometimes

- Often
- Always
- Unsure / I don't know
- Prefer not to say

Please complete the sentence below with the most appropriate answer.

When sugary foods and/or drinks are offered to me I can resist them...

- Strongly disagree
- Disagree
- Neutral / Neither agree nor disagree
- Agree
- Strongly agree
- Unsure / I don't know
- Prefer not to say

Please complete the sentence below with the most appropriate answer.

When there are sugary foods and/or drinks on display I can resist buying them...

- Strongly disagree
- Disagree
- Neutral / Neither agree nor disagree
- Agree
- Strongly agree
- Unsure / I don't know
- Prefer not to say

If I run out of sugary foods and/or drinks I will go out and buy more as soon as possible.

- Never
- Rarely
- Sometimes
- Often
- Always
- Unsure / I don't know

- Prefer not to say

When I eat fewer sugary foods and/or drinks, I have stronger urges to consume them.

- Never
- Rarely
- Sometimes
- Often
- Always
- Unsure / I don't know
- Prefer not to say

I comfort eat sugary foods and/or drinks.

- Strongly disagree
- Disagree
- Neutral / Neither agree nor disagree
- Agree
- Strongly agree
- Unsure / I don't know
- Prefer not to say

I want to eat sugary foods and/or drinks when I feel negative emotions such as anxiety, sadness or depression.

- Strongly disagree
- Disagree
- Neutral / Neither agree nor disagree
- Agree
- Strongly agree
- Unsure / I don't know
- Prefer not to say

I consume sugary foods and/or drinks to ease feelings of anxiety or stress.

- Strongly disagree

- Disagree
- Neutral / Neither agree nor disagree
- Agree
- Strongly agree
- Unsure / I don't know
- Prefer not to say

I consume sugary foods and/or drinks to ease feelings of depression.

- Strongly disagree
- Disagree
- Neutral / Neither agree nor disagree
- Agree
- Strongly agree
- Unsure / I don't know
- Prefer not to say

I have emotional withdrawal symptoms such as anxiety or agitation when I cut down or stop eating sugary foods and/or drinks.

- Strongly disagree
- Disagree
- Neutral / Neither agree nor disagree
- Agree
- Strongly agree
- Unsure / I don't know
- Prefer not to say

I have physical withdrawal symptoms such as a headache, tiredness, or physical pain when I cut down or stop eating sugary foods and/or drinks.

- Strongly disgaree
- Disagree
- Neutral / Neither agree nor disagree
- Agree
- Strongly agree

- Unsure / I don't know
- Prefer not to say

My sugar consumption causes me to feel guilty and yet I can’t cut down on sugary foods and/or drinks.

- Strongly disagree
- Disagree
- Neutral / Neither agree nor disagree
- Agree
- Strongly agree
- Unsure / I don't know
- Prefer not to say

My sugar consumption causes me to feel self-loathing and yet I can’t cut down on sugary foods and/or drinks.

- Strongly disagree
- Disagree
- Neutral / Neither agree nor disagree
- Agree
- Strongly agree
- Unsure / I don't know
- Prefer not to say

My sugar consumption causes me to feel depressed and yet I can’t cut down on sugary foods and/or drinks.

- Strongly disagree
- Disagree
- Neutral / Neither agree nor disagree
- Agree
- Strongly agree
- Unsure / I don't know
- Prefer not to say

My sugar consumption causes me to feel anxiety and yet I can’t cut down on sugary foods and/or drinks.

- Strongly disagree
- Disagree
- Neutral / Neither agree nor disagree
- Agree
- Strongly agree
- Unsure / I don't know
- Prefer not to say

I eat biscuits, cakes, chocolate, sweets, desserts, and other sugary foods.

- Never
- Less than once a month / rarely
- Once a month
- 2-4 times a month
- Once a week
- 2-4 times a week
- 5-6 times a week
- Once a day
- More than once a day
- Unsure / I don't know
- Prefer not to say

I drink beverages with added sugar i.e. full sugar coke (not ‘diet’ alternatives with added sweetener).

- Never
- Less than once a month / rarely
- Once a month
- 2-4 times a month
- Once a week
- 2-4 times a week
- 5-6 times a week

- Once a day
- More than once a day
- Unsure / I don't know
- Prefer not to say

Do you feel stressed regularly (any change or event that causes continuous 'physical, emotional or psychological strain' i.e. work-related stress)?

- Never
- Less than once a month / rarely
- Once a month
- 2-4 times a month
- Once a week
- 2-4 times a week
- 5-6 times a week
- Once a day
- More than once a day
- Unsure / I don't know
- Prefer not to say

I believe my sugar consumption is higher than most other people I know.

- Strongly disgaree
- Disagree
- Neutral / Neither agree nor disagree
- Agree
- Strongly agree
- Unsure / I don't know
- Prefer not to say

When in social occasions, do you persuade others to get a dessert/sweet treat even when they don’t want one, so you can have one?

- Never
- Rarely
- Sometimes

- Often
- Always
- Unsure / I don't know
- Prefer not to say

When eating out, and you want dessert, do you get frustrated at those with you if they don't want a dessert?

- Never
- Rarely
- Sometimes
- Often
- Always
- Unsure / I don't know
- Prefer not to say

I think I am addicted to sugar.

- Strongly disagree
- Disagree
- Neutral / Neither agree nor disagree
- Agree
- Strongly agree
- Unsure / I don't know
- Prefer not to say
- Other (please tell us more in the box below)

Is there anything you would like to add about your sugar consumption? Please type it in the box below (if you have nothing more to add please write "no")?

What age are you? (If you would prefer not to say, please state this).

What is your ethnicity?

- Bangladeshi
- Black African
- Black Caribbean
- Chinese
- Indian
- Middle Eastern
- Mixed
- Pakistani
- White
- Other (please specify)
- Prefer not to say

What is your gender?

- Male
- Female
- Transgender man
- Transgender woman
- Gender neutral
- Non-binary
- Third gender
- Other (please specify)
- Prefer not to say

What is your highest level of education?

- High School Education
- HNC
- HND
- Bachelors Degree
- Honours Degree
- Postgraduate Degree (Masters, MPhil, PhD)

- ◯ [ ] Other (please specify)
- ◯ Prefer not to say

What is your occupation? Please state if you are a student, retired, unemployed or unable able to work.

[ ]

What is your yearly household family income (before tax)?

- ◯ £0
- ◯ £1 to £9,999
- ◯ £10,000 to £24,999
- ◯ £25,000 to £49,999
- ◯ £50,000 to £74,999
- ◯ £75,000 to £99,999
- ◯ £100,000 or more
- ◯ Prefer not to say

Which of the following best describes you (please tick one box only)?

- ◯ Live on my own
- ◯ Live with my spouse/partner with no children
- ◯ Live with my spouse/partner with child(ren)
- ◯ Living with child(ren) as a single parent
- ◯ Living with parent(s)
- ◯ Living in a shared household with other housemate(s) or as a boarder
- ◯ [ ] Other (please specify)

What is your full postcode? (Needed to ensure a broad geographic sample. If you would prefer not to say, please specify).

[ ]

What is your weight (stone and pounds or kilograms)? (If you would prefer not to say or

do not know please specify).

[ ]

What is your height (feet and inches or centimetres)? (If you would prefer not to say or do not know please specify).

[ ]

Do you smoke? Please chose which option applies to you.

- ○ Yes, cigarettes
- ○ I use vapes
- ○ I smoke/use cigarettes and vapes
- ○ No, I don't smoke cigarettes or use vapes
- ○ I am an ex smoker
- ○ I have never smoked
- ○ [ ] Other (please specify)
- ○ Prefer not to say

Do you have any mental (i.e. anxiety, depression) or physical (i.e. asthma, diabetes) health conditions? Please specify which condition(s). If you do not have any conditions, please write 'no', and if you would prefer not to say, please write 'prefer not to say'.

[ ]

Do you have any physical or mental health conditions that may impact eating behaviour i.e. anxiety, eating disorders, diabetes etc? If you do not have any conditions or would prefer not to say, please state this.

[ ]

Do you take any medications that may impact your eating behaviour? If you do not take any medications or prefer not to say, please state this.

[ ]

End

You have reached the end of the questionnaire. Thank you!

Powered by Qualtrics
